# Treatment Satisfaction and Well-Being in Patients with Myopic Choroidal Neovascularization Treated with Ranibizumab in the REPAIR Study

**DOI:** 10.1371/journal.pone.0128403

**Published:** 2015-06-03

**Authors:** Winfried M. Amoaku, Richard P. Gale, Andrew J. Lotery, Geeta Menon, Sobha Sivaprasad, Jennifer Petrillo, Jennifer Quinn

**Affiliations:** 1 University of Nottingham, Academic Ophthalmology, Division of Clinical Neurosciences, and Nottingham University Hospitals NHS Trust, Nottingham, United Kingdom; 2 York Teaching Hospital NHS Foundation Trust, York, United Kingdom; 3 Clinical and Experimental Sciences, Faculty of Medicine, University of Southampton, Southampton, United Kingdom; 4 Frimley Park Hospital NHS Foundation Trust, Frimley, United Kingdom; 5 NIHR Moorfields Biomedical Research Centre, King’s College Hospital NHS Foundation Trust, London, United Kingdom; 6 Novartis Pharma AG, Basel, Switzerland; 7 Novartis Pharmaceuticals UK Limited, Frimley, United Kingdom; Medical College of Soochow University, CHINA

## Abstract

The Ranibizumab for the Treatment of Choroidal Neovascularisation (CNV) Secondary to Pathological Myopia (PM): an Individualized Regimen (REPAIR) trial was a prospective study exploring the efficacy and safety of intravitreal ranibizumab 0.5 mg using an individualized treatment regimen over 12 months. The current study investigated the impact of treatment with ranibizumab as needed (*pro re nata* [PRN]) on individuals with myopic choroidal neovascularization (mCNV) in the REPAIR study, using patient-reported outcome measures (PROMs) for treatment satisfaction and well-being. This study included 65 adults with mCNV and a best-corrected visual acuity (BCVA) letter score of 24–78 in the study eye. Patients completed the Macular Disease Treatment Satisfaction Questionnaire (MacTSQ) at months 1, 6 and 12, and the 12-item Well-Being Questionnaire (W-BQ12) at baseline and months 1, 6 and 12. Subgroup analyses investigated the relationship between PROM scores and treatment in the better- or worse-seeing eye (BSE/WSE), number of injections received, baseline BCVA, BCVA improvement and age. Pearson correlations between change in BCVA, MacTSQ scores and W-BQ12 scores were calculated. The main outcome measures were treatment satisfaction measured with the MacTSQ (score 0–72) and well-being measured with the W-BQ12 (score 0–36). Treatment satisfaction significantly increased over the study period (*p* = 0.0001). Mean MacTSQ scores increased by 9.7 and 10.0 in patients treated in their WSE and BSE, respectively. Treatment satisfaction was highest in individuals receiving only one injection at month 1; however, by month 12, scores were similar across injection subgroups. Patients aged 68 years or older had the highest MacTSQ scores. Well-being scores also significantly increased over the study period (*p =* 0.03). Mean W-BQ12 scores increased by 1.7 in patients treated in their WSE and by 2.1 in patients treated in their BSE. Individuals aged 40 years or younger had the greatest increases in general well-being. Patients who experienced stable or improved BCVA at month 12 had greater increases in W-BQ12 scores than those who experienced a decrease. Correlations between BCVA, MacTSQ scores and W-BQ12 scores were largely non-significant. In conclusion, treatment satisfaction and well-being increased during treatment with ranibizumab PRN. Although directly comparable data are limited for the MacTSQ and W-BQ12 in mCNV, these results complement PROM outcomes reported in related studies.

## Introduction

Pathologic myopia (PM) is a progressive condition characterized by axial elongation and degenerative changes in the posterior segment of the eye [1]. The most prevalent vision-threatening complication of PM, affecting 5–10% of eyes, is myopic choroidal neovascularization (mCNV) [2, 3]. mCNV is responsible for the majority of CNV cases in individuals younger than 50 years [4] and has a poor visual prognosis, with over 90% of affected eyes developing legal blindness, defined as best-corrected visual acuity (BCVA) ≤ 20/200, within 10 years [5, 6]. In patients with unilateral mCNV, approximately 35% will develop mCNV in the fellow eye within 10 years, leading to a significant reduction in visual functioning and health-related quality of life [7].

The main outcomes of trials for retinal diseases tend to focus on visual acuity and safety. However, patient-reported outcomes (PROMs) are increasingly being measured and used to quantify improvement in patient care [8]. Patient satisfaction is an important goal of therapy and can influence adherence to medication and follow-up visits, which is likely to increase treatment success. Patient well-being can be used to assess the emotional impact of visual impairment and the effect of an intervention. Despite the importance of patient satisfaction and well-being, these parameters are not commonly measured in studies of retinal diseases. The only available disease-specific instrument for measuring treatment satisfaction is the Macular Disease Treatment Satisfaction Questionnaire (MacTSQ) [9], which has been used in only one previous published trial [10]. The 12-item Well-Being Questionnaire (W-BQ12) is a short form [11] of the longer parent W-BQ [12–14] and has been shown to be valid for use in individuals with macular disease [15].

The Ranibizumab for the Treatment of Choroidal Neovascularisation Secondary to Pathological Myopia: an Individualized Regimen (REPAIR) trial (ClinicalTrials.gov Identifier: NCT01037348) was a prospective study of the efficacy and safety of intravitreal ranibizumab (Lucentis) 0.5 mg in patients with mCNV using an individualized treatment regimen (n = 65) [16, 17]. The primary outcome measure was the mean change in BCVA from baseline to month 12. In an interim analysis at month 6, the mean improvement in BCVA was 12.2 Early Treatment Diabetic Retinopathy Study (ETDRS) letters [16]. At month 12, a mean improvement of 13.8 letters was achieved using a median of three ranibizumab injections [17]. The study eye had worse visual acuity than the fellow eye in 68% of the cohort [16]. In addition to measuring the change in visual acuity (the primary outcome), REPAIR included the MacTSQ and W-BQ12 as exploratory objectives. These were chosen as appropriate PROMs for treatment with ranibizumab because both treatment satisfaction and well-being are of interest in assessing treatment success.

The present study explored changes in MacTSQ and W-BQ12 scores in individuals treated with ranibizumab in the REPAIR trial. Analyses are presented for MacTSQ and W-BQ12 total and subscale scores, for the overall population and for key patient subgroups.

## Materials and Methods

### Study Design and Patients

The study was conducted in accordance with the Declaration of Helsinki and guidelines for Good Clinical Practice, and approval was obtained from the Sunderland Research ethics committee. Patients provided written informed consent before entering the study. Methodology for the REPAIR trial has been reported previously [16]. In brief, the trial ran from January 2010 to May 2012 and enrolled 65 adults with mCNV and a baseline BCVA score in the study eye of 24–78 ETRDS letters, from 12 centres in the UK. For the primary efficacy variable (change in BCVA from baseline to 12 months), a sample size of 58 was estimated to have 90% power to detect a difference in mean BCVA of 10, assuming a standard deviation of differences of 23, using a paired t-test with a 0.05 two-sided significance level. Sample size calculations were done using nQuery Advisor v6.01. Patients received one initial injection of ranibizumab 0.5 mg followed by repeat injections as needed (*pro re nata* [PRN]). At monthly visits, repeat injections were administered if there was evidence of sub- or intra-retinal fluid on an optical coherence tomography scan, a decrease in BCVA of at least 5 letters or reported increased blurring or metamorphopsia, together with evidence of leakage during fluorescein angiography [16]. The REPAIR trial was not randomized and no control arm was included because the only available comparator, verteporfin, was perceived by clinicians and patients to be inferior to ranibizumab [16].

### Ethics Statement

The study was conducted in accordance with the Declaration of Helsinki and guidelines for Good Clinical Practice, and approval was obtained from the relevant ethics committees. Patients provided written informed consent before entering the study.

### PROM Questionnaires

The MacTSQ was developed to provide a means of evaluating satisfaction with therapies for macular disease [9]. The instrument is based on the Retinopathy Treatment Satisfaction Questionnaire (RetTSQ) [18], with questions specific to diabetic retinopathy being replaced by items important to patients with macular disease [9]. The MacTSQ includes 12 questions, with response options ranging from not at all satisfied (0) to very satisfied (6), generating a range of possible total scores from 0 to 72. The MacTSQ provides two subscale scores: impact of treatment (range 0–36) and information provision and convenience (range 0–36). Higher scores for the total scale and the subscales represent increased satisfaction with treatment.

The W-BQ12 is a 12-item questionnaire [11] that was developed from a 22-item questionnaire designed to measure well-being in individuals with diabetes mellitus [12, 13]. It uses a balance of positively and negatively worded statements addressing the emotional state of the patient with four-point scales – from not at all (0) to all the time (3) – to provide a general well-being score. It provides three subscale scores: negative well-being (range 0–12), energy (range 0–12) and positive well-being (range 0–12). The W-BQ12 general well-being score is calculated by reversing the negative well-being score (reversed range 12–0) and summing with the energy and positive well-being scores [15]. General well-being scores range from 0 to 36, with higher values representing greater well-being. The psychometric properties of the W-BQ12 have been investigated in patients with macular disease, and the instrument has been shown to have the same factor structure as obtained previously in patients with diabetes. Good reliability has been demonstrated for both the general well-being score and the subscale scores [15].

Patients completed the PROM questionnaires during study visits, before visiting a physician or receiving treatment. The MacTSQ was completed at months 1, 6 and 12 and the W-BQ12 was completed at baseline and months 1, 6 and 12.

### Statistical Analysis

All analyses were conducted using the full analysis set (all patients who received at least one injection and who had at least one post-baseline assessment) and the last-observation-carried-forward approach. Paired t-tests were used to test mean differences over time. To evaluate the relationship between BCVA, treatment satisfaction and well-being, Pearson and Spearman correlations between change in BCVA, MacTSQ scores and W-BQ12 scores were calculated, demonstrating no significant differences. When comparing the strength of the correlations, *r* values less than 0.30 were regarded as weak, 0.30–0.50 as moderate and more than 0.50 as strong. Because PROMs were exploratory objectives in the REPAIR study, the reported results should be interpreted as descriptive – no α correction for multiplicity was conducted. All statistical analyses were performed using SAS software version 9.3.

*Subgroup analyses*: Analyses were conducted to investigate PROM scores when the study eye was the better-seeing eye (BSE) or worse-seeing eye (WSE), defined by a baseline visual acuity better or worse than that of the fellow eye by ≥ 5 letters, respectively, for patients with baseline BCVA ≥ 50 letters (approximate Snellen equivalent, better than 20/100) in both eyes, or of ≥ 10 letters for those with baseline BCVA < 50 letters [19]. Subgroup analyses were conducted to determine the relationship between PROM scores and the number of injections received (one, two or three, and more than three), baseline BCVA (≤ 52, 53–67, ≥ 68; LogMAR [Snellen equivalent] worse than 0.66 [20/100^+^], 0.65–0.36 [20/80^-^–20/50^+^], better than 0.34 [20/40^-^]), BCVA improvement (< 0 letters, 0–4 letters, 5–9 letters, ≥ 10 letters) and patient age (< 40, 40–68 and > 68 years).

## Results

### Patient Demographics and Baseline Characteristics

In total, 65 patients with mCNV were recruited, with 62 completing the study. Patient demographics and baseline characteristics are described in **Table 1**. Most participants were women (70.8%), the majority (66%) were aged 40–68 years, and 91% were Caucasian. The majority of participants (68%) received treatment in their WSE. Individuals received a median of three injections over the 12-month study period (mean 3.6).

**Table 1:** Patient demographics and baseline characteristics.

Characteristic	Subgroup	Overall population (N = 65)
**Age, years (mean ± SD)**		55.5 ± 15.0
**Age, years (n, %)**	< 40 years	11 (17)
	40–68 years	43 (66)
	> 68 years	11 (17)
**Female (n, %)**		46 (71)
**Caucasian (n, %)**		59 (91)
**Weight, kg (mean ± SD)**		76.8 ± 18.0
**PM duration, years (mean ± SD)**		39.9 ± 20.5
**CNV duration, months (mean ± SD)**		1.8 ± 3.3
**BCVA, letters (mean ± SD)**		59.5 ± 13.6
**BCVA, letters (n, %)**	< 52	20 (30.8)
	53–67	25 (38.5)
	≥ 68	20 (30.8)
**CRT, μm (mean ± SD)**		384.7 ± 130.9

BCVA, best-corrected visual acuity; BSE, better-seeing eye; CNV, choroidal neovascularization; CRT, central retinal thickness; PM, pathologic myopia; SD, standard deviation; WSE, worse-seeing eye.

### Treatment Satisfaction

**Fig 1:**
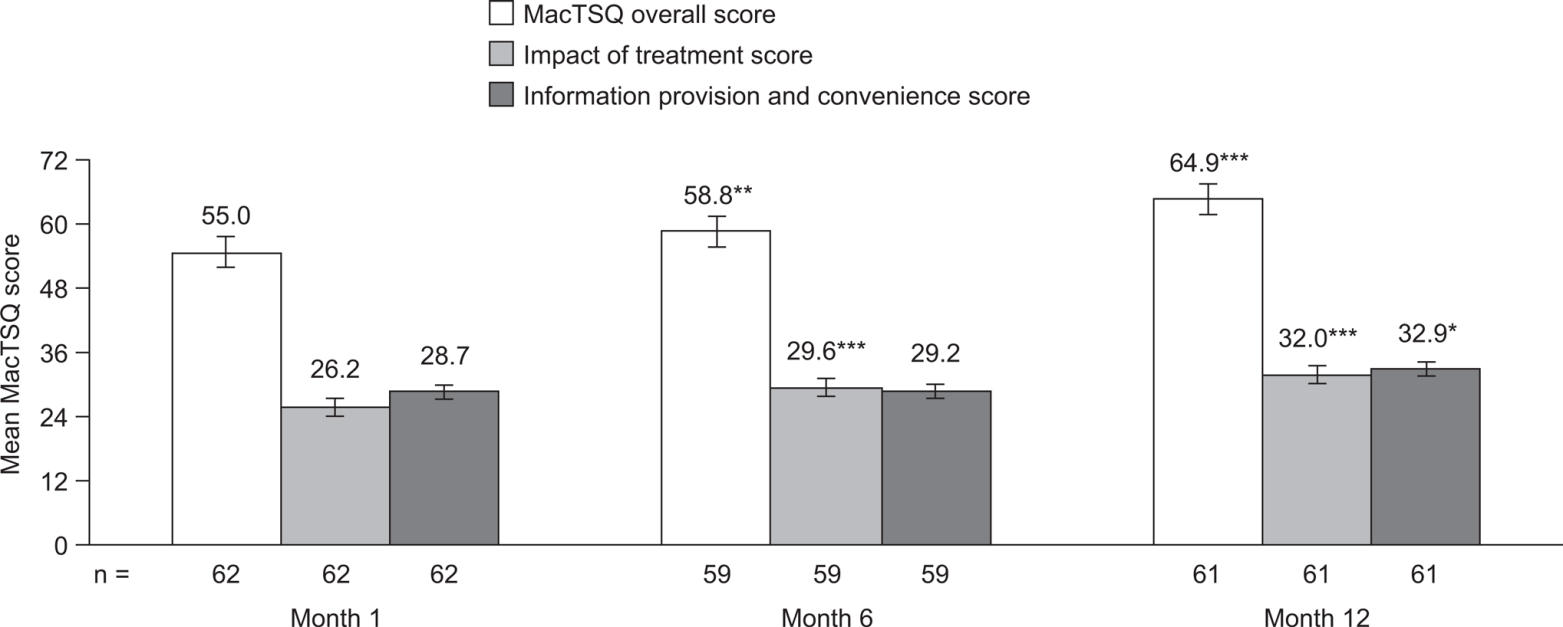
Mean MacTSQ total score and subscale scores. Data are shown as mean ± standard error. *p<0.05 vs month 1. **p<0.01 vs month 1. ***p<0.001 vs month 1. MacTSQ, Macular Disease Treatment Satisfaction Questionnaire.

**Fig 2:**
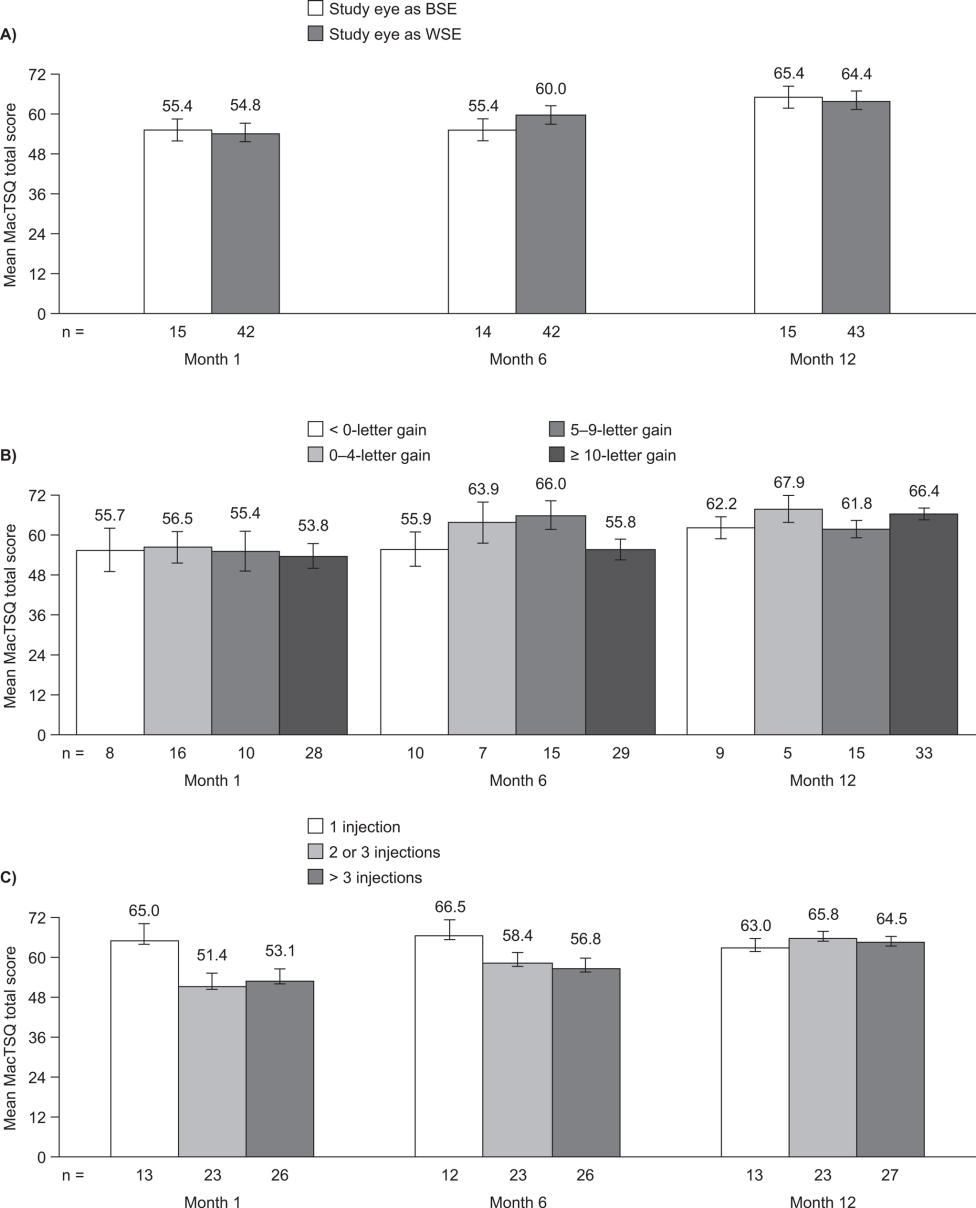
MacTSQ total scores. (A) BSE/WSE, (B) BCVA improvement and (C) number of injections. Data are shown as mean ± standard error. BCVA, best-corrected visual acuity; BSE, better-seeing eye; MacTSQ, Macular Disease Treatment Satisfaction Questionnaire; WSE, worse-seeing eye.

At month 1, patients reported a mean total MacTSQ score of 55.0, which increased to 58.8 at month 6 (*p =* 0.005) and 64.9 at month 12 (*p =* 0.0001) (Fig 1). Similar increases were seen in both MacTSQ subscales; the impact of treatment subscale score was 26.2 at month 1, which increased to 29.6 at month 6 (*p*<0.001) and 32.0 at month 12 (*p*<0.001), and the information provision and convenience subscale score was 28.7 at month 1, which increased to 29.2 at month 6 (*p =* 0.55) and 32.9 at month 12 (*p =* 0.014). The total MacTSQ score did not change from month 1 to month 6 when the study eye was the BSE (55.4 for both time points), but increased from 54.8 to 60.0 when the study eye was the WSE (Fig 2A). The corresponding scores at month 12 were 65.4 and 64.4. The increases in scores in the BSE and WSE subgroups were similar for each of the MacTSQ subscales.

There were no statistically significant differences in treatment satisfaction among patients of different ages at baseline, although those aged over 68 years had numerically higher MacTSQ total scores than younger patients (**Table 2**). Individuals with a baseline BCVA of 52 letters or fewer had the highest mean MacTSQ total score at all time points (**Table 2**). All baseline BCVA subgroups, however, had similar increases in treatment satisfaction from month 1 to month 12. No significant differences in mean MacTSQ total scores were seen among BCVA improvement subgroups, with similar levels of treatment satisfaction in all subgroups at month 12 (Fig 2B). Patients requiring only the initial injection had the highest overall treatment satisfaction at months 1 and 6 (Fig 2C). At month 12, those receiving two or three injections reported the highest MacTSQ total scores. There were no significant differences in scores among injection number subgroups at any time. Unfortunately some patients were unable to complete the relevant questionnaires consistently at every visit, resulting in variations in numbers in Fig 2.

**Table 2:** MacTSQ results according to baseline BCVA and age.

Characteristic	Subgroup	MacTSQ total score		
		Month 1	Month 6	Month 12
**Baseline BCVA, letters**	≤ 52 (n = 20)	59.2 ± 3.5^a^	66.3 ± 1.7^b^	67.9 ± 1.7^a^
	53–67 (n = 25)	51.7 ± 4.1^b^	54.6 ± 3.9^b^	62.7 ± 2.2^b^
	≥ 68 (n = 20)	54.8 ± 4.0	56.8 ± 4.1^a^	64.7 ± 2.1^a^
**Age, years**	< 40 (n = 11)	53.7 ± 5.4	62.4 ± 4.8	61.3 ± 2.9
	40–68 (n = 41) ^b^	53.2 ± 2.8	56.7 ± 2.5	65.3 ± 1.5
	> 68 (n = 11)	63.6 ± 5.7^a^	67.0 ± 5.3^b^	65.5 ± 2.9

Data are shown as mean ± standard error.

^a^Data missing for one patient.

^b^Data missing for two patients.

BCVA, best-corrected visual acuity; MacTSQ, Macular Disease Treatment Satisfaction Questionnaire.

### Well-Being

**Fig 3:**
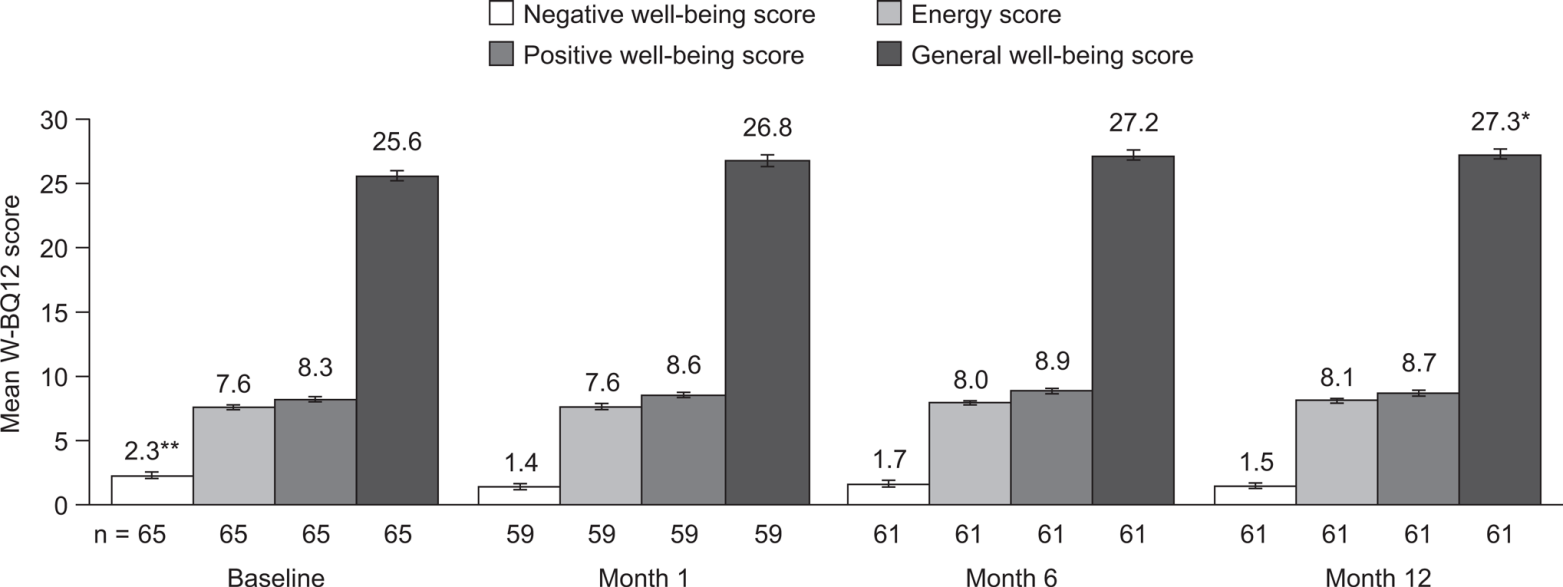
Mean W-BQ12 negative well-being, energy, positive well-being and general well-being scores. Data are shown as mean ± standard error. *p<0.05 vs baseline. **p<0.01 vs baseline. W-BQ12, 12-item Well-Being Questionnaire.

**Fig 4:**
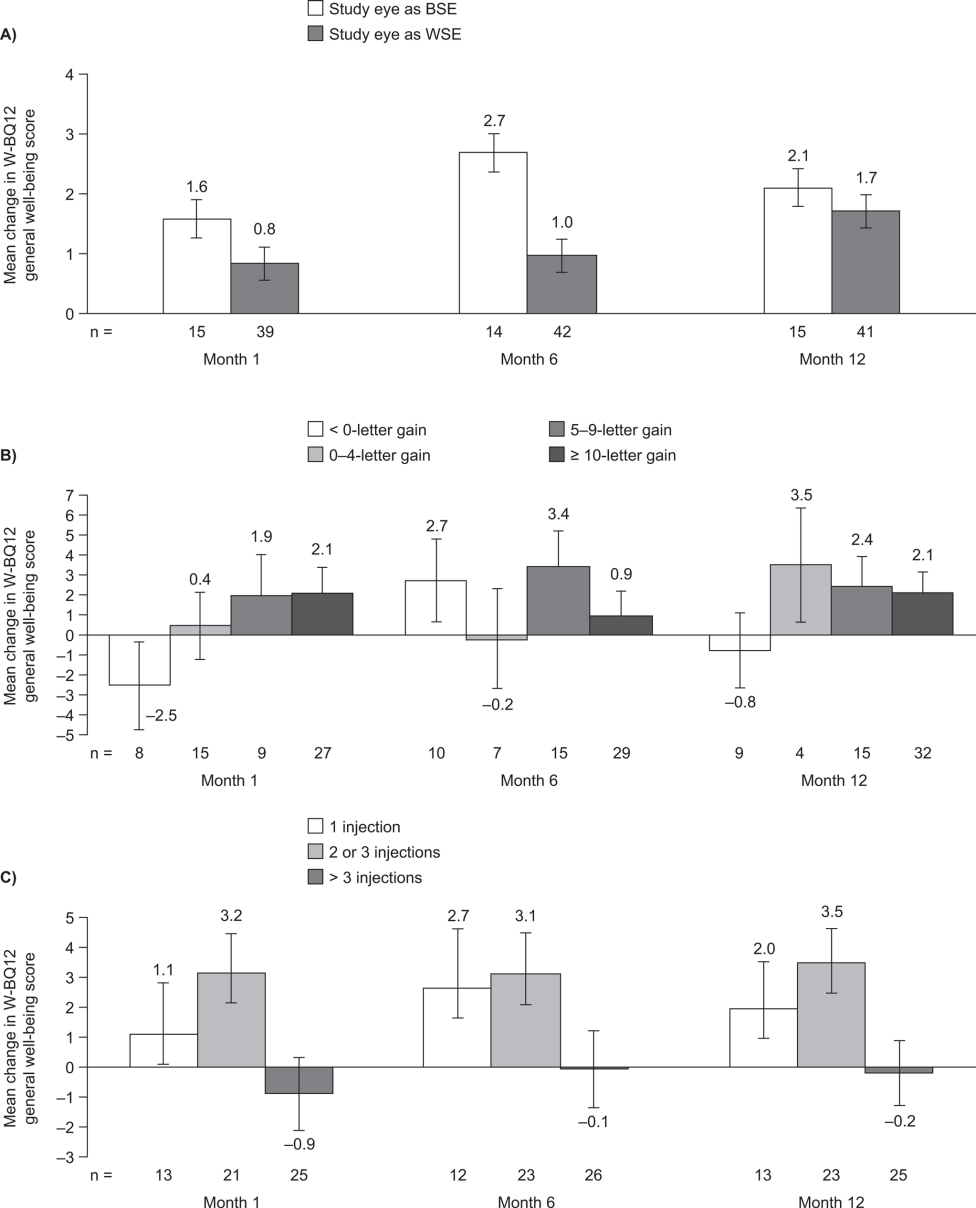
Mean change in W-BQ12 general well-being scores. (A) BSE/WSE, (B) BCVA improvement and (C) number of injections. Data are shown as mean ± standard error. BCVA, best-corrected visual acuity; BSE, better-seeing eye; W-BQ12, 12-item Well-Being Questionnaire; WSE, worse-seeing eye.

The mean W-BQ12 general well-being score at baseline was 25.6. General well-being was maintained at months 1 and 6 (26.8 and 27.2, respectively), and significantly increased from baseline to month 12 (25.6 vs 27.3; *p =* 0.03) (Fig 3). At month 12 there were numerical improvements in all W-BQ12 subscale scores, but these did not reach statistical significance. When the study eye was the BSE, the general well-being, energy and positive well-being scores increased by 2.7, 0.4 and 0.1, respectively, from baseline to month 6 (Fig 4A). The corresponding score increases from baseline to month 12 were 2.1, 0.4 and 0.2. The W-BQ12 negative well-being score decreased by 2.1 from baseline to month 6, and by 1.5 from baseline to month 12, in the BSE subgroup. When the study eye was the WSE, the W-BQ12 negative well-being score decreased from baseline to month 12 by 0.5. W-BQ12 general well-being, energy, and positive well-being scores increased by 1.0, 0.3 and 0.7, respectively, from baseline to month 6 in the WSE subgroup. The corresponding score increases from baseline to month 12 were 1.7, 0.5 and 0.8.

Baseline W-BQ12 scores were similar across all subgroups, although scores were slightly higher in patients with a baseline BCVA of 52 letters or fewer than in the other baseline BCVA subgroups, and in patients treated in their WSE rather than in their BSE (S1 Table, available online). There were no statistically significant differences in the change from baseline in W-BQ12 general well-being scores among patients of different ages, although those aged 39 years and younger at baseline had the highest numerical increase from baseline to month 12. Patients with a baseline BCVA of 52 letters or fewer reported a slight decrease in mean W-BQ12 general well-being score of 0.2 from baseline to month 12, compared with increases in general well-being scores in those with a baseline BCVA of at least 53 letters (**Table 3**). Patients who experienced stable or improved BCVA at month 12 had greater increases in W-BQ12 general well-being scores than those who had a decrease in BCVA (Fig 4B). The number of injections received did not significantly affect changes in W-BQ12 general well-being scores, although the greatest increase from baseline to month 12 was reported by patients receiving two or three injections (mean change, +3.49) (Fig 4C).

**Table 3:** W-BQ12 results according to baseline BCVA and age.

Characteristic	Subgroup	Change in W-BQ12 score from baseline		
		Month 1	Month 6	Month 12
**Baseline BCVA, letters**	≤ 52 (n = 20)	-0.8 ± 0.9^c^	-0.3 ± 1.5^b^	-0.2 ± 1.2^a^
	53–67 (n = 25)	2.5 ± 1.5^c^	2.1 ± 1.3	2.1 ± 1.2^b^
	≥ 68 (n = 20)	0.9 ± 1.8	3.1 ± 1.7^b^	2.9 ± 1.5^a^
**Age, years**	< 40 (n = 11)	2.8 ± 2.0^a^	1.1 ± 2.0	4.0 ± 1.9^b^
	40–68 (n = 41)^b^	1.0 ± 1.0^b^	1.7 ± 1.1	1.5 ± 0.9
	> 68 (n = 11)	-0.8 ± 2.0^a^	2.5 ± 2.2^b^	0.3 ± 1.7

Data are shown as mean ± standard error.

^a^Data missing for one patient.

^b^Data missing for two patients.

^c^Data missing for three patients.

BCVA, best-corrected visual acuity; W-BQ12, 12-item Well-Being Questionnaire.

### Correlations between BCVA, MacTSQ and W-BQ12

Correlations between change in BCVA, MacTSQ total score and W-BQ12 general well-being score were weak at all time points assessed (month 12, *r =* 0.02 for both correlations). Correlations between MacTSQ total score and W-BQ12 general well-being score were also weak (month 12, *r =* 0.08) (S2 Table, available online). Strong correlations were found between total score and subscale scores for each PROM, but subscale scores correlated only weakly across instruments (S3 Table, available online).

## Discussion

While the MacTSQ has been used previously in a recent study of neovascular age-related macular degeneration (nvAMD), to the best of our knowledge, this is the first study to report the effect of a specific treatment for mCNV on patients’ well-being and satisfaction with treatment [10]. The results demonstrate that individuals with mCNV treated with ranibizumab PRN for up to 12 months in the REPAIR trial experienced high overall treatment satisfaction, measured by the MacTSQ, and that satisfaction increased during the study period. Patients also reported a modest but statistically significant improvement in general well-being over the study period, measured by the W-BQ12. Individuals whose visual acuity deteriorated during the study had negative changes in W-BQ12 scores and generally had the lowest gains in MacTSQ scores, confirming the validity of the two instruments in real practice. Benchmark data for clinically significant changes in people with macular disease are limited for both the MacTSQ and the W-BQ12. For both instruments, the results of individual subscales were generally similar to those of the overall scales.

Intraocular injections may be associated with anxiety among patients, and the increases observed over time in MacTSQ scores may reflect habituation to treatment regimen. Individuals in the REPAIR trial received individualized treatment with injections PRN, and the increase in treatment satisfaction over the study period suggest that patients understand that they can achieve lasting visual acuity gains when injection frequency is tailored in this way.

The increases in MacTSQ total scores, impact of treatment subscale scores and information and convenience subscale scores were similar in the BSE and WSE subgroups, although at month 6 patients treated in their WSE had slightly higher scores than those treated in their BSE. Baseline W-BQ12 scores were slightly higher in patients treated in their BSE than in patients treated in their WSE, possibly reflecting better overall binocular vision of patients treated in their BSE. Patients with baseline BCVA of 52 letters or fewer also had higher baseline W-BQ12 scores; this may be a result of the higher expectations from treatment for these patients, although it may be a chance finding. Overall the differences between subgroups in both the MacTSQ and the W-BQ12 were minor and therefore would be unlikely to affect the final results; nevertheless, it may be useful to adjust for this in future studies. The increases in the W-BQ12 and MacTSQ scores at 12 months compared with baseline values were slightly greater in patients treated in their BSE than in those treated in their WSE. This is in agreement with the results of previous studies: across a number of ocular diseases and instruments, scores from PROM instruments such as the National Eye Institute 25-item Visual Functioning Questionnaire (NEI VFQ-25) and health utility scores (e.g. as measured using the five-dimension EuroQoL questionnaire) have consistently been found to be associated with visual acuity in the BSE [20–26].

At month 1, patients requiring one injection over the 12-month study period had higher treatment satisfaction than those requiring two or three injections or more than three injections, perhaps reflecting treatment benefits achieved between the initial injection and the month 1 visit, which meant that another injection was not required according to the REPAIR protocol. At month 12, MacTSQ scores were similar among subgroups receiving one, two or three or more than three injections, suggesting that treatment satisfaction is independent of injection number. Mean changes in W-BQ12 scores were not significantly different among injection subgroups, but were numerically higher in individuals receiving one to three injections than in those receiving more than three injections. At months 1 and 6, patients over 68 years had numerically higher levels of treatment satisfaction than younger participants; this difference may be because retired individuals can more conveniently attend daytime clinics. All subgroups, however, reported similar levels of treatment satisfaction at the end of the study. Low BCVA at baseline was associated with the highest levels of treatment satisfaction, although there was no clear relationship between change in BCVA and MacTSQ scores.

The results of the correlation analysis showed that there was only a weak correlation between BCVA and MacTSQ scores, and between BCVA and W-BQ12 scores. There was little correlation between the two PROM instruments, confirming that the MacTSQ and W-BQ12 measure distinctly different clinical concepts.

There are no comparable data from other studies for the MacTSQ and W-BQ12 in patients with mCNV. The fact that the MacTSQ has previously been employed in only one study [9] and therefore has not been rigorously tested in multiple investigations represents a limitation in the current study. Nevertheless, the MacTSQ is the only retinal disease-specific instrument available to measure treatment satisfaction and thus was adopted here. A recent study for nvAMD, the IVAN study (Inhibition of Vascular Endothelial Growth Factor in Age-Related Choroidal Neovascularization), used the MacTSQ and reported treatment satisfaction scores similar to those seen in the present study, although detailed MacTSQ analyses from the IVAN study have yet to be published [10]. Our PROM results are complemented by the findings of the RADIANCE study (Ranibizumab and Photodynamic Therapy Evaluation in Myopic Choroidal Neovascularization) [27]. The RADIANCE study included assessments using the widely used NEI VFQ-25, which measures patient-reported vision-related functioning across 11 subscales. The NEI VFQ-25 results demonstrated greater improvements in vision-related functioning in patients with mCNV treated with ranibizumab than in those treated with verteporfin [27].

Although the REPAIR trial is the second largest published study investigating ranibizumab for the treatment of visual impairment due to mCNV (following the RADIANCE study) [27], the number of patients enrolled is limited. The MacTSQ and W-BQ12 results showed considerable variability over time, particularly in the analyses of patient subgroups, and the small number of individuals in each subgroup limits the statistical power of the analyses to detect differences in outcomes for particular subgroups. It is also notable that most patients (68%) received treatment in their WSE, which may lead to lower increases in these measures than might be expected for patients treated in the BSE. In addition, as mentioned previously, the REPAIR trial was not randomized and no control arm was included, as the only available comparator, verteporfin, was deemed inferior to ranibizumab. Nevertheless, the sustained improvements in NEI VFQ-25 scores reported in the randomized RADIANCE trial, presented at the 13th European Society of Retina Specialists Congress, support the findings of REPAIR [27].

In conclusion, these results show that patients experience increases in treatment satisfaction and well-being following treatment with ranibizumab PRN, which complements the visual acuity outcomes reported previously and the vision-related functioning outcomes reported in RADIANCE [16, 17, 27]. In addition, these data provide a benchmark for MacTSQ and W-BQ12 results for future studies of intravitreal therapies for mCNV. These PROMs appear to measure distinct clinical concepts and reflect parameters that are overlooked by existing visual function instruments commonly used in clinical trials of macular treatments. Inclusion of the MacTSQ (or the related RetTSQ) and W-BQ12 instruments in future clinical trials in patients with mCNV and other retinal diseases, in addition to existing visual function [28] and quality of life [29, 30] measures, could therefore provide valuable information on the outcomes of treatment. The results of this study show that the MacTSQ and W-BQ12 are likely to be sensitive even when patient numbers are modest, although larger sample sizes would have provided greater confidence in the findings from the subgroup analyses.

## Supporting Information

S1 Table Baseline W-BQ12 scores. (DOCX)

S2 Table Pearson correlations between change in BCVA, MacTSQ scores and W-BQ12 scores. (DOCX)

S3 Table Correlations between change in BCVA and MacTSQ and W-BQ12 scores. (DOCX)
