# Supplementary material for: Treatment Satisfaction and Well-Being in Patients with Myopic Choroidal Neovascularization Treated with Ranibizumab in the REPAIR Study
Source: PLoS One. 2015 Jun 3;10(6):e0128403. doi: 10.1371/journal.pone.0128403 (PMC4454435; doi:10.1371/journal.pone.0128403)
Supplement: S1 Table — (DOCX) [file pone.0128403.s001.docx]

**Treatment Satisfaction and Well-being in Patients With Myopic Choroidal Neovascularization Treated With Ranibizumab in the REPAIR Study**

Winfried M. Amoaku^1*^, Richard P. Gale^2^, Andrew J. Lotery^3^, Geeta Menon^4^, Sobha Sivaprasad^5^, Jennifer Petrillo^6^, Jennifer Quinn^7^

^1^University of Nottingham, Academic Ophthalmology, Division of Clinical Neurosciences, and Nottingham University Hospitals NHS Trust, Nottingham, United Kingdom

^2^York Teaching Hospital NHS Foundation Trust, York, United Kingdom

^3^Clinical and Experimental Sciences, Faculty of Medicine, University of Southampton, Southampton, United Kingdom

^4^Frimley Park Hospital NHS Foundation Trust, Frimley, United Kingdom

^5^NIHR Moorfields Biomedical Research Centre, King’s College Hospital NHS Foundation Trust, London, United Kingdom

^6^Novartis Pharma AG, Basel, Switzerland

^7^Novartis Pharmaceuticals UK Limited, Frimley, United Kingdom

* Corresponding author

E-mail: [Winfried.Amoaku@nottingham.ac.uk](mailto:Winfried.Amoaku@nottingham.ac.uk) (WMA)

Table S1. Baseline W-BQ12 scores.

| **Characteristic** | **Subgroup** | **Baseline W-BQ12** **score** | | | |
| --- | --- | --- | --- | --- | --- |
|  |  | **Energy** | **Positive well-being** | **Negative well-being** | **General well-being** |
| Study eye | BSE (n = 15) | 6.6 < 2.7 | 7.9 < 3.1 | 3.3 < 3.4 | 23.2 < 7.8 |
|  | Neither BSE or WSE (n = 6) | 6.3 < 3.6 | 6.0 < 3.6 | 4.8 < 3.5 | 19.5 < 10.5 |
|  | WSE (n = 44) | 8.1 < 2.2 | 8.7 < 2.4 | 1.6 < 2.1 | 27.2 < 5.4 |
| Baseline BCVA, letters | ≤ 52 (n = 20) | 8.1 < 2.1 | 9.4 < 1.8 | 1.2 < 1.5 | 28.3 < 4.2 |
|  | 53–67 (n = 25) | 7.0 < 2.7 | 7.8 < 2.9 | 3.2 < 3.5 | 23.5 < 7.6 |
|  | ≥ 68 (n = 20) | 7.9 < 2.7 | 7.8 < 3.1 | 2.2 < 2.5 | 25.5 < 7.8 |
| Age, years | < 40 (n = 11) | 7.8 < 2.8 | 8.3 < 3.0 | 2.5 < 3.2 | 25.6 < 7.1 |
|  | 40–68 (n = 41) | 7.7 < 2.5 | 7.9 < 2.8 | 2.4 < 2.8 | 24.8 < 7.4 |
|  | >68 (n = 11) | 9.6 < 1.7 | 8.6 < 2.2 | 1.6 < 2.4 | 28.6 < 4.0 |
| BCVA improvement to month 12, letter gain | < 0 (n = 8) | 7.0 < 3.0 | 7.3 < 3.5 | 2.9 < 3.5 | 23.4 < 9.4 |
|  | 0–4 (n =16) | 8.8 < 2.9 | 7.8 < 4.2 | 3.0 < 3.1 | 25.6 < 9.9 |
|  | 5–9 (n =10) | 7.3 < 2.5 | 7.9 < 3.2 | 2.7 < 3.2 | 24.6 < 7.7 |
|  | ≥ 10 (n =28) | 7.7 < 2.5 | 8.8 < 2.1 | 1.8 < 2.5 | 26.6 < 5.6 |
| Number of injections | 1 (n = 13) | 7.6 < 2.8 | 7.9 < 3.4 | 1.9 < 2.7 | 25.6 < 8.1 |
|  | 2–3 (n = 23) | 7.3 < 2.0 | 7.7 < 2.5 | 2.9 < 2.7 | 24.1 < 5.9 |
|  | >3 (n = 27) | 7.8 < 2.8 | 9.0 < 2.5 | 2.0 < 3.0 | 26.8 < 7.2 |

Data are shown as mean < standard deviation. BCVA, best-corrected visual acuity; BSE, better-seeing eye; W-BQ12, 12-item Well-Being Questionnaire; WSE, worse-seeing eye.
