# Supplementary material for: Treatment Satisfaction and Well-Being in Patients with Myopic Choroidal Neovascularization Treated with Ranibizumab in the REPAIR Study
Source: PLoS One. 2015 Jun 3;10(6):e0128403. doi: 10.1371/journal.pone.0128403 (PMC4454435; doi:10.1371/journal.pone.0128403)
Supplement: S2 Table — (DOCX) [file pone.0128403.s002.docx]

**Treatment Satisfaction and Well-being in Patients With Myopic Choroidal Neovascularization Treated With Ranibizumab in the REPAIR Study**

Winfried M. Amoaku^1*^, Richard P. Gale^2^, Andrew J. Lotery^3^, Geeta Menon^4^, Sobha Sivaprasad^5^, Jennifer Petrillo^6^, Jennifer Quinn^7^

^1^University of Nottingham, Academic Ophthalmology, Division of Clinical Neurosciences, and Nottingham University Hospitals NHS Trust, Nottingham, United Kingdom

^2^York Teaching Hospital NHS Foundation Trust, York, United Kingdom

^3^Clinical and Experimental Sciences, Faculty of Medicine, University of Southampton, Southampton, United Kingdom

^4^Frimley Park Hospital NHS Foundation Trust, Frimley, United Kingdom

^5^NIHR Moorfields Biomedical Research Centre, King’s College Hospital NHS Foundation Trust, London, United Kingdom

^6^Novartis Pharma AG, Basel, Switzerland

^7^Novartis Pharmaceuticals UK Limited, Frimley, United Kingdom

* Corresponding author

E-mail: [Winfried.Amoaku@nottingham.ac.uk](mailto:Winfried.Amoaku@nottingham.ac.uk) (WMA)

**Table S2. Pearson correlations between change in BCVA, MacTSQ scores and W-BQ12 scores.**

| **Time point** |  | **BCVA change from baseline** | **MacTSQ total score** | **W-BQ12 general well-being score** |
| --- | --- | --- | --- | --- |
| **Month 1** | BCVA change from baseline | 1.00 |  |  |
|  | MacTSQ total score | 0.12 | 1.00 |  |
|  | W-BQ12 general well-being score | 0.11 | 0.07 | 1.00 |
| **Month 6** | BCVA change from baseline | 1.00 |  |  |
|  | MacTSQ total score | −0.05 | 1.00 |  |
|  | W-BQ12 general well-being score | −0.26 | 0.14 | 1.00 |
| **Month 12** | BCVA change from baseline | 1.00 |  |  |
|  | MacTSQ total score | 0.02 | 1.00 |  |
|  | W-BQ12 general well-being score | 0.02 | 0.09 | 1.00 |

Data are shown as *r*.

BCVA, best-corrected visual acuity; MacTSQ, Macular Disease Treatment Satisfaction Questionnaire; W‑BQ12, 12-item Well-Being Questionnaire.
