# Supplementary material for: Treatment Satisfaction and Well-Being in Patients with Myopic Choroidal Neovascularization Treated with Ranibizumab in the REPAIR Study
Source: PLoS One. 2015 Jun 3;10(6):e0128403. doi: 10.1371/journal.pone.0128403 (PMC4454435; doi:10.1371/journal.pone.0128403)
Supplement: S3 Table — (DOCX) [file pone.0128403.s003.docx]

**Treatment Satisfaction and Well-being in Patients With Myopic Choroidal Neovascularization Treated With Ranibizumab in the REPAIR Study**

Winfried M. Amoaku^1*^, Richard P. Gale^2^, Andrew J. Lotery^3^, Geeta Menon^4^, Sobha Sivaprasad^5^, Jennifer Petrillo^6^, Jennifer Quinn^7^

^1^University of Nottingham, Academic Ophthalmology, Division of Clinical Neurosciences, and Nottingham University Hospitals NHS Trust, Nottingham, United Kingdom

^2^York Teaching Hospital NHS Foundation Trust, York, United Kingdom

^3^Clinical and Experimental Sciences, Faculty of Medicine, University of Southampton, Southampton, United Kingdom

^4^Frimley Park Hospital NHS Foundation Trust, Frimley, United Kingdom

^5^NIHR Moorfields Biomedical Research Centre, King’s College Hospital NHS Foundation Trust, London, United Kingdom

^6^Novartis Pharma AG, Basel, Switzerland

^7^Novartis Pharmaceuticals UK Limited, Frimley, United Kingdom

* Corresponding author

E-mail: [Winfried.Amoaku@nottingham.ac.uk](mailto:Winfried.Amoaku@nottingham.ac.uk) (WMA)

Table S3. Correlations between change in BCVA and MacTSQ and W-BQ12 scores.

| **Time point** |  | **Change in BCVA from baseline** | **MacTSQ score** | | | **W-BQ12** | | |
| --- | --- | --- | --- | --- | --- | --- | --- | --- |
|  |  |  | **Information provision and convenience** | **Impact of treatment** | **Total** | **Negative well-being** | **Energy** | **Positive well-being** |
| **Month 1** | Change in BCVA from baseline | 1.00 |  |  |  |  |  |  |
|  | MacTSQ information provision and convenience | 0.05 | 1.00 |  |  |  |  |  |
|  | MacTSQ impact of treatment | 0.19 | 0.82* | 1.00 |  |  |  |  |
|  | MacTSQ total | 0.12 | 0.97* | 0.94* | 1.00 |  |  |  |
|  | W-BQ12 negative well-being | −0.02 | −0.11 | −0.15 | −0.13 | 1.00 |  |  |
|  | W-BQ12 energy | 0.19 | 0.12 | 0.15 | 0.14 | −0.51* | 1.00 |  |
|  | W-BQ12 positive well-being | 0.06 | −0.04 | −0.06 | −0.05 | −0.49* | 0.58* | 1.00 |
|  | W-BQ12 general well-being | 0.11 | 0.06 | 0.08 | 0.07 | −0.76* | 0.84* | 0.88* |
| **Month 6** | Change in BCVA from baseline | 1.00 |  |  |  |  |  |  |
|  | MacTSQ information provision and convenience | −0.05 | 1.00 |  |  |  |  |  |
|  | MacTSQ impact of treatment | −0.04 | 0.81* | 1.00 |  |  |  |  |
|  | MacTSQ total | −0.05 | 0.96* | 0.94* | 1.00 |  |  |  |
|  | W-BQ12 negative well-being | 0.18 | 0.04 | −0.04 | 0.01 | 1.00 |  |  |
|  | W-BQ12 energy | −0.29* | 0.06 | 0.17 | 0.12 | −0.55* | 1.00 |  |
|  | W-BQ12 positive well-being | −0.18 | 0.17 | 0.28* | 0.23 | −0.41* | 0.64* | 1.00 |
|  | W-BQ12 general well-being | −0.26* | 0.08 | 0.20 | 0.14 | −0.79* | 0.87* | 0.84* |
| **Month 12** | Change in BCVA from baseline | 1.00 |  |  |  |  |  |  |
|  | MacTSQ information provision and convenience | 0.02 | 1.00 |  |  |  |  |  |
|  | MacTSQ impact of treatment | 0.02 | 0.32* | 1.00 |  |  |  |  |
|  | MacTSQ total | 0.02 | 0.88* | 0.74* | 1.00 |  |  |  |
|  | W-BQ12 negative well-being | −0.14 | −0.06 | −0.14 | −0.11 | 1.00 |  |  |
|  | W-BQ12 energy | −0.06 | 0.17 | 0.09 | 0.17 | −0.42* | 1.00 |  |
|  | W-BQ12 positive well-being | −0.04 | −0.04 | −0.05 | −0.05 | −0.27* | 0.47* | 1.00 |
|  | W-BQ12 general well-being | 0.02 | 0.08 | 0.08 | 0.09 | −0.74* | 0.80* | 0.76* |

**p* < 0.05. BCVA, best-corrected visual acuity; MacTSQ, Macular Disease Treatment Satisfaction Questionnaire; W-BQ12, 12-item Well-Being Questionnaire.
